# Computational Simulations in Advanced Microfluidic Devices: A Review

**DOI:** 10.3390/mi12101149

**Published:** 2021-09-23

**Authors:** Violeta Carvalho, Raquel O. Rodrigues, Rui A. Lima, Senhorinha Teixeira

**Affiliations:** 1 MEtRICs, Campus de Azurém, University of Minho, 4800-058 Guimarães, Portugal; rl@dem.uminho.pt; 2 ALGORITMI, Campus de Azurém, University of Minho, 4800-058 Guimarães, Portugal; st@dps.uminho.pt; 3 Center for MicroElectromechanical Systems (CMEMS-UMinho), Campus de Azurém, University of Minho, 4800-058 Guimarães, Portugal; raquel.rodrigues@dei.uminho.pt; 4 CEFT, R. Dr. Roberto Frias, Faculty of Engineering of the University of Porto (FEUP), 4200-465 Porto, Portugal

**Keywords:** computational simulations, drug discovery, organ-on-a-chip, microfluidic devices, preclinical models, numerical simulations

## Abstract

Numerical simulations have revolutionized research in several engineering areas by contributing to the understanding and improvement of several processes, being biomedical engineering one of them. Due to their potential, computational tools have gained visibility and have been increasingly used by several research groups as a supporting tool for the development of preclinical platforms as they allow studying, in a more detailed and faster way, phenomena that are difficult to study experimentally due to the complexity of biological processes present in these models—namely, heat transfer, shear stresses, diffusion processes, velocity fields, etc. There are several contributions already in the literature, and significant advances have been made in this field of research. This review provides the most recent progress in numerical studies on advanced microfluidic devices, such as organ-on-a-chip (OoC) devices, and how these studies can be helpful in enhancing our insight into the physical processes involved and in developing more effective OoC platforms. In general, it has been noticed that in some cases, the numerical studies performed have limitations that need to be improved, and in the majority of the studies, it is extremely difficult to replicate the data due to the lack of detail around the simulations carried out.

## 1. Introduction

Over the years, the demand for more reliable and effective preclinical models has grown as we try to reduce and, where possible, replace the utilization of animal models for assessing drug efficacy and safety [1]. Although animal models provide a physiological structure similar to that of human cells and organs, they fail to adequately forecast the behavior of and represent human metabolism. These experiments also require laborious protocols, present ethical issues, and are expensive [2,3,4,5]. Due to the low throughput of in vivo testing, researchers have been using advanced microfluidic devices that integrate biomodels to replicate the physiology and function of cells and/or organs. A microfluidic device is the building block of the microfluidic chip family, defined as a set of technologies embedded in a chip that contains microchannels with a dimension of tens to hundreds of micrometers to process a small volume of fluids. These microdevices first emerged mainly for analytical purposes, and due to the small amount of reagents and sample requirements, they attracted increasing interest thanks to their several advantages over other standard technologies, such as the short time needed for analysis, reduction in fabrication and reagent costs, miniaturization, selectivity, sensitivity, portability, and biocompatibility. In time, the ability to integrate multiple processes in a single device led to the concept of lab-on-a-chip or micro total analysis systems (µTASs), which were first introduced by Andreas Manz in 1990 [6,7]. Their versatility enables their application in a variety of fields, from environmental monitoring and the food industry and microelectronics to biomedical or clinical applications. 

Their biocompatibility and ability to precisely study, evaluate, or diagnose individual or multiple cells in a dynamic and accessible way brought forth the concept of cells-on-a-chip. In these biodevices, cells are inserted (mixed with an organic fluid) into the microfluidic chip, which allows assessing their unique function and behavior outside of the body, maintaining their integrity and biological functionality. 

Lastly, and more recently, another concept has been introduced: organ-on-a-chip (OoC), also known as microphysiological systems. In these advanced microfluidic devices, 3D cell structures containing mono or multiple cell lines are embedded in an extracellular matrix to emulate the complexity and architecture of the mimicked living organ [8,9]. These devices allow more complex studies and analyses which are physiologically closer to what we can find in a human body system. In these organ-on-a-chip devices, studies can be devoted to single organs and functions, but also to the interaction and response of multiple organs, where the ultimate goal is a body-on-a-chip which is able to replicate the entire human body in a single platform [10,11]. Among the several attributes of these models, their ability to accelerate research, accurately reproduce the micro-environment of human cells, and be mass-produced in a cost-effective way should be stressed [12,13]. Nevertheless, there are also certain drawbacks associated with them, which have to be overcome over time. Frequently, polydimethylsiloxane (PDMS) is used in the fabrication of biomedical and advanced microfluidic devices owing to its outstanding properties, including optical transparency, permeability to gases, and biocompatibility [14,15,16,17,18,19,20,21,22]. However, small hydrophobic molecules, such as biomolecules, proteins, and drugs, can be adsorbed to the surface and consequently compromise the quality of the experiments’ analysis in OoC platforms [23,24,25]. Nonetheless, as the advantages of these devices exceed their limitations, these models continue to be a valuable tool in the study of new health treatments. Many researchers have developed several OoCs to simulate the function of the heart [26], lungs [27], liver [28], kidney [29], brain [30], bone [31], etc., or a combination of several organs, known as body-on-a-chip [32]. The last one allows a better understanding of not only the efficacy of a drug, but also its potential toxicity in other organs, which in turn guarantees a better validity of the results and extrapolation to reality [33]. In addition to the previous models, recently, Si et al. [34] used a human-airway-on-a-chip to investigate the antimalarial drug amodiaquine as a powerful inhibitor of infection with SARS-CoV-2. The research team modeled a human bronchial airway epithelium and pulmonary endothelium infected with pseudotyped severe acute respiratory syndrome coronavirus 2 and verified that the antimalarial drug amodiaquine has the potential to inhibit infection. Hence, the use of OoC helped in the rapid advances witnessed in the testing of new drugs to fight the new coronavirus, which has become an urgent need for the fight against the COVID-19 pandemic.

Along with the progress made in the development of new microphysiological systems, computational tools have played an important role. These allow complementing experimental studies, from an engineering point of view, allowing us to study physical phenomena, assess the viability of a device, and optimize the design at a lower cost [35,36,37,38,39]. Furthermore, their capability to determine critical parameters which are difficult to measure via experimental methods, such as pressure, velocity, shear rate, and temperature, constitute other benefits of using numerical methods. For these reasons, researchers have complemented their studies with numerical methods [40,41,42]. Although a substantial number of review papers exist in the field of microfluidic OoC-based systems, only a small number have addressed the different approaches using numerical simulations as an auxiliary tool in the development of these devices [11,35,37,43]. Hence, the present work aims to provide an overview of the most recent progress in numerical studies in advanced microfluidic devices and how researchers have applied computational tools in their investigations.

## 2. Applications of Numerical Simulations in Advanced Microfluidic Devices

In this section, the usefulness of conducting numerical simulations in parallel with experimental studies is presented, namely, in the study of fluid flow and mass transfer, nanoparticle development, and also in the optimization of advanced microfluidic devices, i.e., OoC and cell-on-a-chip.

### 2.1. Fluid Flow and Mass Transfer

A key factor that affects the functionality of OoC platforms is fluid flow, which includes the evaluation of several parameters, such as velocity fields, shear stress, and the concentration of vital parameters for cellular function, such as oxygen, glucose, and temperature. 

**Figure 1:**
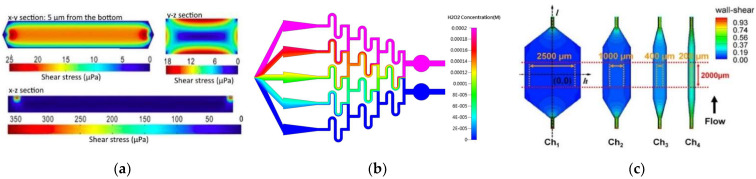
(**a**) CFD simulation of shear stress on the XY, YZ, and ZX planes. Adapted from [45]; (**b**) numerical simulation of $H_{2}O_{2}$ concentration inside the microfluidic chip. Adapted from [46]; (**c**) contours of wall shear stress distribution in designed chambers. From left to right, central areas of each chamber, with different horizontal distances. The orange and red dashed lines indicate the uniform wall shear stress area boundary. Adapted from [47].

The flow of the culture medium is of great importance to maintain an adequate amount of nutrients for the cell culture as well as the shear stresses exposed to cells, and therefore, understanding flow behavior is vital in the design of OoC microfluidic devices [44]. Kocal et al. [45] simulated flow behavior in a cancer microenvironment to understand the uniformity of fluid shear stress along with microfluidic devices. The results showed that adherent cancer cells experienced a homogenous wall shear stress of up to 25 μPa in the microchannels (Figure 1a). Another interesting study was conducted by Lo et al. [46]. They investigated the influence of shear stress and antioxidant concentrations on the production of reactive oxygen species in lung cancer cells by combining experimental and numerical simulations, and analogous results were obtained for both methods. They verified that an increase in shear stress leads to an increase in the production of reactive oxygen species (Figure 1b). Additionally, two antioxidants, α-tocopherol and ferulic acid, were tested to verify their ability to reduce reactive oxygen species. It was found that a high dose of α-tocopherol was not able to eliminate reactive oxygen species, but a lower dose could. The same outcome was not observed for ferulic acid. This antioxidant could eliminate the production of reactive oxygen species in a concentration-independent way. Kou et al. [47], on the other hand, developed a microfluidic system with the ability to create four different shear flows in only one device to study the cytosolic calcium concentration dynamics of osteoblasts. They observed that cytosolic calcium concentration in the osteoblasts increased proportionally to the intensity of shear stress from 0.03 to 0.30 Pa (Figure 1c). Komen et al. [48] developed a microfluidic device with the ability to expose cancer cells to an in-vivo-like concentration profile of a drug and measure the effectiveness on-chip. This device comprised a cell culture chamber and a drug-dosing channel separated by a transparent membrane so that the drug is not exposed to shear stresses and also to allow a label-free growth quantification. They simulated cell exposure and verified that it followed the blood concentrations measured in vivo.

**Figure 2:**
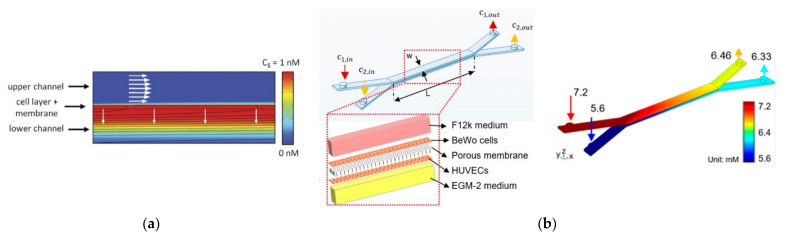
(**a**) Computational modeling of fluid flow and analyte transport in microchannels and porous membrane. White arrows indicate flow direction in the membrane and the upper microfluidic channel. The upper surface of the cell layer has a fixed concentration equal to 1 nM. Analyte flux streamlines are shown in black. Adapted from [36]; (**b**) chip design and glucose concentration profiles at a flow rate of 50 µL/h in the bare membrane, respectively. Adapted from [51].

The diffusion of species is also of utmost importance for cell maintenance, and porous zones are commonly modeled to study these phenomena. Wong et al. [36] created a computational model of a microfluidic device that incorporates flow-induced shear stress and monitors cell-secreted biomolecules. For that, they modeled a bilayer device in which cells are cultured on a porous membrane support. This way, researchers can adjust the device to operate with physiological shear stresses, while ensuring the rapid transport of secreted biomolecules to the biosensor (Figure 2a). In another study [49], Wong et al. used the same model but to simulate the concentration of electroactive tracers, which they validated with experiments. A similar model was proposed by Chen et al. [50], consisting of a porous membrane-separated and a microfluidic chip channel with two layers. In their study, they evaluated the dependences of flow features on the chip geometry and membrane permeability using immersed boundary methods—IBM. The results presented elucidated the dependences of flow flux, wall shear stress, and transmembrane pressure difference on the chip geometry (channel height and length) and membrane permeability, which were consistent with the experimental results. For a membrane with lower permeability, a lengthier channel was required to achieve a permeability-independent stage. Moreover, it was also observed that wall shear stress was dependent on channel length and membrane permeability. Mosavati et al. [51] developed a numerical model of a three-dimensional placenta-on-a-chip model with a porous medium to model the membrane separating the two channels (Figure 2b). The researchers aimed to analyze the unsteady flow into microchannels and glucose concentration profiles in different locations of the microfluidic chip. After validation of the model, Mosavati et al. studied the effects of flow rate and membrane porosity on glucose diffusion through the placental barrier. Similarly, in a recent study [4], Banaeiyan et al. explored the diffusion of glucose, since this is the main element present in the culture medium, through diffusion channels. By contrast, Hu and Li [52] numerically investigated oxygen concentration in the interior of a three-dimensional microchannel containing tumor spheroids and tested two different velocities, 50 µm/s and 100 µm/s. They observed that with lower velocity, oxygen concentration downstream of the spheroid surface is not enough for spheroid growth. By raising the perfusion velocity, oxygen concentration increases and may consequently enhance the growth of the spheroids. Another model comprising a permeable layer was presented by Zahorodny-Burke and Elias [53]. Briefly, they developed a rectangular channel with a polymer layer permeable to oxygen linked to a glass substrate seeded with one layer of oxygen-consuming cells. In contrast, Bhise and coworkers [54] did not simulate a porous membrane where oxygen passes through it. Instead, they modeled hydrogels as a porous medium with a homogenous volumetric oxygen consumption rate related to the total number of encapsulated cells. Other researchers have studied not only the diffusion of species but also the convection of carbon dioxide—CO_2_ [55]. A computational model was developed to investigate CO_2_ transport in a cylindrical microfluidic device. The level of CO_2_ at the base of the water chamber for different CO_2_ supplying concentrations was investigated, and it was found that by increasing the level of CO_2_ in the feeding gas to above 10%, CO_2_ concentration at the bottom of the chamber reaches the desired level of 5%. 

The study of the effects of radiation treatment on microcirculation was recently addressed by Cicchetti et al. [56]. The authors developed a numerical model to understand the effects of radiation therapy on normal tissues surrounding a tumor. They modeled the vasodilation and variation of membrane permeability as inputs with consequent fluid extravasation and the deposition of fibrin or the formation of edema. In a second simulation, the researchers modeled the impact of variations in vessel wall elasticity on the distribution of blood flow velocity.

**Figure 3:**
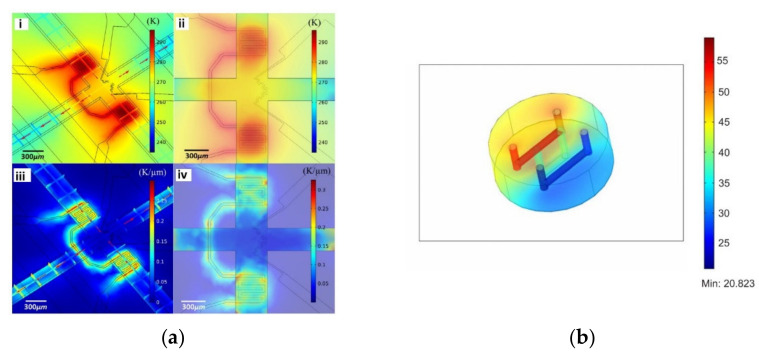
(**a**) Numerical simulation of on-chip temperature profiles including spatial temperature distribution (i), planar temperature distribution (ii), spatial temperature gradient distribution (iii), and planar temperature gradient distribution (iv). Adapted from [58]; (**b**) temperature distribution in the chip in the incubator environment (370 K ambient and bath at 900 K). Adapted from [59].

Another important parameter in these types of studies is temperature because it affects not only the environment but also the behavior and function of cells [57]. Hence, its monitoring is of great importance for cell culture. Peng et al. [58] conducted a multiphysics simulation (fluid flow and heat transfer) in a microfluidic device to obtain an accurate and rapid control of on-chip local temperature control. According to the results, the device was able to operate with temperatures ranging between 2 °C and 37 °C using cooling and heating components (Figure 3a). A similar study was conducted by Das and coworkers [59], in which a microfluidic device to study the viability and activity of the cells under a temperature gradient was developed. Through numerical simulations, the temperature gradient that can be reached in the proposed chip was assessed, and it was found that the proposed chip is capable of creating temperature conditions that realistically mimic physiological/biological conditions (Figure 3b).

**Figure 4:**
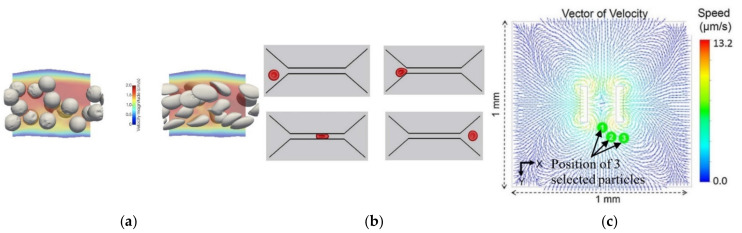
(**a**) Simulation of relatively stiff beads with a shear elastic modulus, Gs, of 10−3 N/m, compared to deformable cells Gs = 10−5 N/m. Adapted from [60]; (**b**) deformation of an RBC passing through a narrow microchannel. Adapted from [61]; (**c**) vector of velocity on the XY plane and position of the selected particles in experimental tests. Adapted from [63].

Although single-phase simulations are mostly used, the use of multiphase models is a valuable option to improve the precision of simulations and obtain more detailed information. To study the behavior of circulating tumor cells in a 3D bioprinted vasculature chip, Hynes et al. [60] performed numerical simulations using both the lattice Boltzmann method (LBM) and finite element method (Figure 4a). The particles were treated as either rigid or deformable spheres, and the results matched closely to the experimental findings, proving that the numerical model used was appropriate. By contrast, Ye et al. [61] used a smoothed dissipative particle dynamics (SDPD) method to model the fluid flow and an IBM for the fluid–cell interaction to study the RBC mechanics in a microfluidic chip (Figure 4b). Likewise, Zhang et al. [62], in order to simulate mechanical interactions between flow and particles (cells) for a cells-on-a-chip device, used a two-way Euler/Lagrange multiphase model. Another example of the use of multiphase models in this type of study was presented by Tanaka et al. [63]. The authors developed a hybrid pump driven by cardiomyocytes that self-organize into bridges between elastic micro-pier structures (Figure 4c). In addition, they performed numerical simulations to understand the flow pattern and distribution of flow velocity, and the results were in good agreement with the experimental results. They used the volume of fluid (VOF) model to better understand and verify the flow generation, flow pattern, and velocity distribution of the ultra-small fluid oscillation unit. 

**Figure 5:**
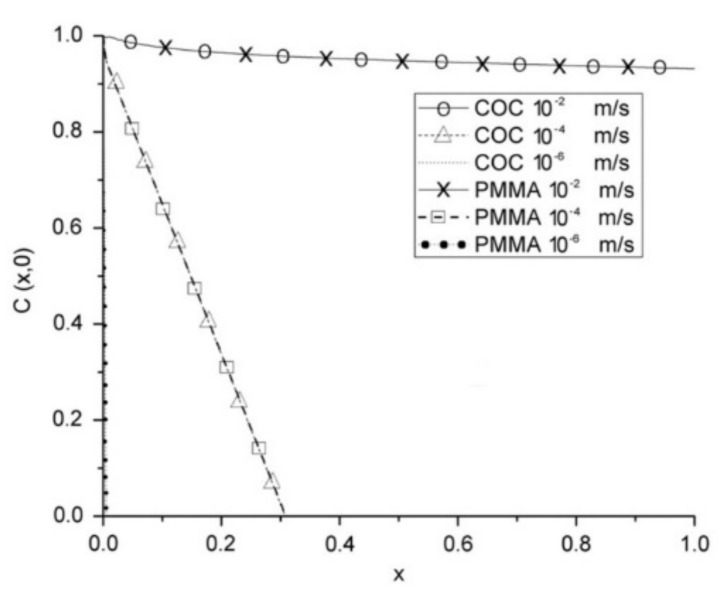
COC and PMMA at various average flow rates with a polymer capping layer with a thickness of 1.9 mm. At this thickness, for corresponding speeds, both polymers have virtually identical profiles. Adapted from [53].

Another key parameter that affects the function of a device is the material. As previously mentioned, PDMS is preferred due to its suitable properties and low price [5,23,64]. Moreover, this material can be obtained with different air permeability, which is interesting for improving the diffusion of species in advanced microfluidic devices. Lamberti et al. [65] demonstrated that by increasing the PDMS mixing ratio, air permeability can be improved up to 300% when compared to the standard 10:1 ratio. However, this material also has certain limitations that encourage researchers to seek alternative materials, one of them being the possibility of absorption of small hydrophobic species [23,64]. Two alternative materials are poly(-methylmethacrylate) (PMMA) [66,67,68,69] and cyclic olefin copolymer (COC) [70]. Zahorodny-Burke et al. [53] studied the nature of oxygen transport within microfluidic cell culture devices considering PDMS, COC, and PMMA through a two-dimensional convection–diffusion mass transfer model. To this end, flow rate, diffusive layer thickness, and material selection were investigated in order to determine their relation to ensure adequate supplies of oxygen for cell growth. However, special attention must be paid when using COC or PMMA. Due to the low oxygen diffusion of these materials, flow rate must be optimized to ensure a sufficient supply of oxygen to the cells by applying high flow rates, at an order of 10^−2^ m/s (Figure 5). By contrast, Ochs and coworkers [71] investigated not only PDMS and COC, but also a different type of material, polymethylpentene (PMP). They experimentally and numerically studied the influence of PMP on oxygen concentration in cell culture and verified that this is a possible alternative for PDMS. PMP was shown to be able to deliver sufficient oxygen for various types of cells, namely, hepatocytes with a high oxygen consumption rate. 

### 2.2. Nanoparticle Simulations

Nanoparticles (NPs) have been attracting interest for their potential use as drug delivery systems, but the optimization of their physical properties, such as their size and shape, is a point of interest to accomplish appropriate cell responses [72,73,74]. Due to the multidisciplinarity of computational software, several researchers have taken advantage of its capabilities to broaden the understanding of the transport of NPs and drugs in OoC [75,76,77,78,79]. Soheili et al. [75] performed a numerical and experimental study on the microfluidic preparation and production of chitosan NPs. To develop NPs with the desired size, they implemented a model that relates NPs’ diameter to the flow rate, and sodium tripolyphosphate (TPP) concentration as a critical factor that affects cell flow behavior. Further, the researchers found the optimal mixing efficiency of tripolyphosphate conditions by associating TPP concentration with the length of the channels and flow rate. Kwak and coworkers [79] developed a tumor-microenvironment-on-chip to recapitulate the key features of complex transport of drugs and NPs within a tumor microenvironment. By combining computational simulations and experimental tests, they obtained more detailed information about the dynamic transport behavior of NPs and concluded that NPs should be conceived considering their interactions with the tumor microenvironment.

**Figure 6:**
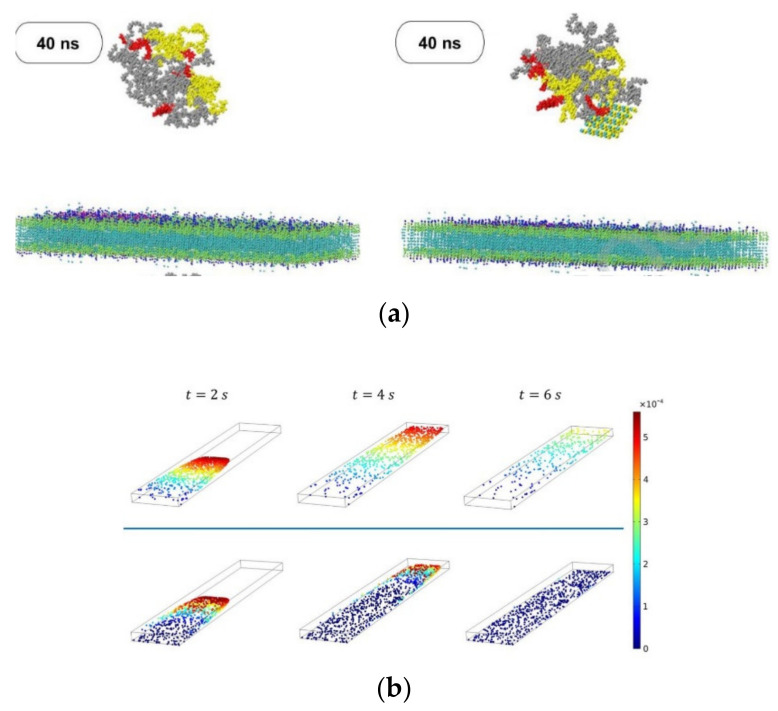
(**a**) Nanocarrier penetration into the cancer cell membrane without MoSe_2_ and with MoSe_2_, respectively. Adapted from [78]; (**b**) Distribution and instantaneous velocity of non-Brownian particles at t = 2, 4 and 6 s for particle diameter d = 100 nm and 500 nm. Adapted from [76].

Other authors have been motivated by the “catch and release” of biomolecules observed in physiological processes and used numerical simulations to design synthetic systems that can catch, transport, and release targeted species inside the surrounding solution, which can be useful for effective separation processes within microfluidic devices [77]. Their setup allows the determination of the conditions where the oscillating fins of the device can selectively bind and ‘‘catch’’ target NPs in the upper channel, and then release them in the lower channel. Their findings have provided insights into fabrication devices to detect and separate target molecules from complex fluids. This topic has been addressed by various researchers [80,81,82,83]. Maleki and coworkers [78] developed a hybrid drug carrier of doxorubicin (DOX) due to the high consumption rate of riboflavin in cancerous cells. In parallel, molecular simulations to study the effects of the microfluidic environment and shift metal dichalcogenide nanolayers on the stability, size, and self-assembly interaction energies of the nanocarriers were conducted (Figure 6a). The results showed that among the studied configurations, the most suitable ones for the adsorption of DOX molecules are MoSe_2_, MoSSe, MoO_3_, and MoS_2_, respectively. Furthermore, the appearance of the new coronavirus has triggered several scientific investigations, and the study conducted by Arefi and colleagues [76] was one of them. They studied the effect of airborne NPs on human health through both numerical simulations and experimental work. Transport and adsorption in a microfluidic lung-on-a-chip device were investigated, and two different multiphase models were used. For the fine NPs, particle transport was estimated using an Eulerian advection-diffusion approach, while for coarse NPs, the Lagrangian particle tracking approach was used (Figure 6b). One of their findings showed that during physical exercise, particle deposition increases, and thus, outdoor exercise on days with poor air quality should be reduced.

## 3. Numerical Optimization

**Figure 7:**
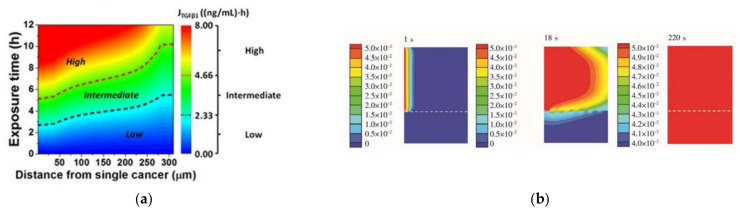
(**a**) Contour plot of the TGFβ1 exposure ($J_{\mathrm{TGF}\beta 1}$) in (distance, exposure time)-space, where the numerical simulation was conducted. Adapted from [84]; (**b**) glucose concentration profile at different instants (1, 18, and 220 s) in the chip. Adapted from [85].

The use of numerical simulations to optimize devices is one of the main steps that must be carried out during the design process. This helps researchers to improve and accelerate the development of more efficient and representative models for a given problem by subjecting the chip to design changes and improvements. Experimental conditions can also be optimized in accordance with numerical simulations. For instance, Karakas and coworkers [84] developed a microfluidic chip for screening individual cancer cells and performed numerical simulations in order to optimize the exposure time for autophagy, a cellular mechanism where proteins are consumed and recycled to give another source of energy to cells (Figure 7a). This numerical study allowed investigators to determine the minimum exposure time required to ensure the viability of the experiments, saving time and resources during experimental tests. On the other hand, Zhang et al. [81] studied the effect of varying the inlet flow rate of blood and buffer on the separation performance of circulating tumor cells in a microfluidic chip and optimized the operating conditions to increase separation efficiency. Another way to improve a device’s accuracy was presented by Jun-Shan et al. [85], who developed a microfluidic chip with micropillar arrays for 3D cell culture, and using numerical simulations, the space between micropillars was optimized, which allowed the nutrients in the medium to quickly diffuse into the chamber, while cell metabolites could diffuse out of the chamber in a timely manner (Figure 7b). A similar study with micropillars was performed by Chen and coworkers [86]. However, in this case, the authors studied pillars with circular, elliptical, and square cross-sections assembled in both aligned and staggered patterns. Through numerical simulations, the researchers found that in the latter case, fluid flows through both the center of the array and around the pillars. These observations led the research team to address this approach since in staggered arrangements, fluid is better distributed throughout the device. Zhang et al. [62] used a two-way Euler/Lagrange multiphase model to simulate mechanical interactions between flow and particles (cells) for cells-on-a-chip devices. The authors developed three different designs and studied the effect of using different cell densities, inlet flow velocities, and inlet cell numbers. The results showed that regions of lower strain rates had high cell concentrations, and when lower inlet velocities (10 and 20 µm/s) were used, high cell concentrations were observed. However, they observed that cells were not able to reach the outlet, while with a velocity of 40 µm/s, some cells were.

Optimization methods are also of great importance for discovering the relationship between several variables. For instance, Huang and Nguyen [87] simulated a cell-stretching device and optimized important dimensional parameters. They performed a parametrical optimization to determine the relationship between the lateral displacement of the membrane and wall height, wall thickness, membrane thickness, and vacuum strength in order to maximize the output strain of the stretched membrane.

In Table 1, a summary of the numerical considerations used in the previously reviewed papers is presented, including the software used, the flow behavior, flow type, fluid rheology, mesh, flow analysis, and also the validation.

A brief analysis of the results reveals several blank spaces which represent the absence of information regarding the assumptions made when performing the numerical works. Additionally, the preferred software for these types of studies was found to be COMSOL Multiphysics^®^ software, followed by Ansys software^®^, although researchers have begun to develop their own simulation software to overcome some of the limitations of the existing ones [56,60]. Moreover, a fundamental step in numerical simulations is mesh development. Without guaranteeing that the mesh is appropriate, one cannot obtain truthful and accurate results. Hence, both mesh quality and independence have to be ensured, and this is a step that is commonly missing in the studies presented in this review article. Some authors conducted mesh independence studies, but the majority only indicate the number of elements or do not present any information. One way to carry out such studies with more accuracy is through the calculation of the grid convergence index, as represented in the study conducted by Soheili et al. [75], increasing, in this way, the reliability of the presented results. Moreover, it can be seen that the validation of these types of studies is usually disregarded, but this is an extremely important step that should be addressed whenever possible.

## 4. Final Remarks and Future Directions

Advanced microfluidic devices have revolutionized drug development processes and in vitro tests by allowing to save time and money but also, more importantly, to present a preclinical platform that can accurately mimic human tissues/organs and thus diminish the use of animals in clinical testing. These devices have become research accelerators, therefore rapidly achieving the status of a promising technology, which has now been proven to be useful for advances in emerging diseases, such as research related to the new coronavirus, Sars-Cov-2, and cancer treatment. Nevertheless, although this technology has been shown to be extremely valuable, it is still far from being able to precisely mimic a proper human organ, which has hindered the passage of these devices to the market. Therefore, research and development of more complex and reliable advanced microfluidic devices will continue to progress for several years, and numerical simulations are expected to boost this process by not only allowing mass transport insights, but also reducing the costs and saving time in research. Hence, computational methods are envisioned to become a fundamental tool for the advance of research in advanced microfluidic platforms. 

The present review intended to encourage current and future researchers to include this tool in their studies, and it has shown the importance of using numerical simulations in parallel with experimental works to improve the performance and quality of advanced microfluidic devices. Nevertheless, it was found that most numerical studies did not include details about the simulations used, which makes it unfeasible for other researchers to recreate such simulations and understand what methods were actually used to obtain certain outputs. 

In addition to the advantages of using numerical tools, there are also certain challenges and limitations associated with numerical modeling platforms. As biomedical devices become more complicated, mathematical modeling platforms need further improvement and validation to become more precise, and this is a lengthy and difficult process. Moreover, in these biological studies, there are a lot of physical and chemical processes occurring both at a micro- and macro-scale, and it becomes difficult to assess and represent these in one numerical model, making simulations extremely complex and time-consuming. However, given the advancements in this type of technology, the field is expected to grow by leaps and bounds, and more biological features will be included in the existing software. For instance, molecular dynamics simulations may be one good alternative to perform more complex simulations. 

## Figures and Tables

**Table 1:** Summary of the considerations made in the numerical studies.

Software	Flow Regime	Phases Number	Fluid Rheology	Mesh	Time- Dependence	Validation	Ref
COMSOL	Laminar and turbulent	Single-phase	Newtonian	n/d	Steady	×	[45]
CFD-ACE +	Laminar	Single-phase	n/d	n/d	Steady	✓	[46]
Ansys	Laminar	Single-phase	Newtonian	78,000 nodes	Steady	×	[47]
COMSOL	n/d	Single-phase	n/d	n/d	Transient	✓	[48]
COMSOL	Laminar	Single-phase	Newtonian	182,900 elements	Transient	×	[36]
n/d	Laminar	Single-phase	n/d	n/d	Transient	✓	[49]
IBM, MATLAB and Ansys	Laminar	Multiphase	Newtonian	Mesh independence test (1 µm)	Transient	×	[50]
COMSOL	Laminar	Single-phase	Newtonian	Mesh independence test (n/d)	Transient	✓	[51]
COMSOL	Laminar	Single-phase	Newtonian	Fine mesh (n/d)	Transient	×	[4]
COMSOL	n/d	Single-phase	n/d	n/d	Transient	✓	[52]
COMSOL	Laminar	Single-phase	n/d	7000 elements	Steady	✓	[53]
COMSOL	n/d	Single-phase	n/d	Mesh independence test (800,000 nodes)	Transient	×	[54]
COMSOL	n/d	Multiphase	Newtonian	24,300 elements	Transient	✓	[55]
User-Defined Software	Laminar	Single-phase	n/d	n/d	Steady	×	[56]
COMSOL	Laminar	Single-phase	n/d	n/d	Steady	×	[58]
COMSOL	Laminar	Single-phase	n/d	123,334 elements	Steady	✓	[59]
User-Defined Software	n/d	Multiphase	Newtonian	n/d	Transient	✓	[60]
SDPD and IBM	n/d	Multiphase	n/d	n/d	Transient	✓	[61]
Ansys	Laminar	Multiphase	Newtonian	Mesh independence test (220,000 elements)	Transient	✓	[62]
Ansys	Laminar	Multiphase	Newtonian	n/d	Steady	✓	[63]
COMSOL	n/d	Single-phase	n/d	$1.8\times 10^{6}$ elements	Transient	✓	[71]
COMSOL	Laminar	Multiphase	n/d	Mesh independence test (6000 elements)	Transient	×	[75]
n/d	n/d	Multiphase	n/d	n/d	Transient	✓	[79]
n/d	n/d	Multiphase	n/d	n/d	Transient	×	[77]
GROMACS	n/d	Multiphase	Newtonian	n/d	Transient	✓	[78]
COMSOL	Laminar	Multiphase	n/d	Mesh independence test (168,000 elements)	Steady and Transient	✓	[76]
COMSOL	n/d	Single-phase	n/d	n/d	Transient	×	[84]
n/d	n/d	Multiphase	n/d	n/d	Transient	×	[81]
COMSOL and Ansys	n/d	Single-phase	n/d	n/d	Transient	✓	[85]
Ansys	n/d	Single-phase	n/d	n/d	Steady	×	[86]
Ansys	n/d	Single-phase	n/d	2–8 μm element size	Transient	✓	[87]

n/d—non-defined.
